# Giant coronary aneurysm in a toddler with Kawasaki disease: technical challenges in CT coronary angiography

**DOI:** 10.1093/bjrcr/uaad008

**Published:** 2023-12-18

**Authors:** Yan Hei Chan, Catherine Yee Man Young, Ki Wang, Enoch C T So, Winnie C W Chu

**Affiliations:** Department of Imaging and Interventional Radiology, Faculty of Medicine, The Chinese University of Hong Kong, Shatin, New Territories, Hong Kong; Department of Imaging and Interventional Radiology, Faculty of Medicine, The Chinese University of Hong Kong, Shatin, New Territories, Hong Kong; Department of Imaging and Interventional Radiology, Faculty of Medicine, The Chinese University of Hong Kong, Shatin, New Territories, Hong Kong; Department of Paediatrics, Faculty of Medicine, The Chinese University of Hong Kong, Shatin, New Territories, Hong Kong; Department of Imaging and Interventional Radiology, Faculty of Medicine, The Chinese University of Hong Kong, Shatin, New Territories, Hong Kong

**Keywords:** kawasaki disease, giant coronary artery aneurysm, CTCA

## Abstract

Kawasaki disease is the most common vasculitis causing acquired coronary artery aneurysm (CAA) and affects mostly children. Computed tomography coronary angiography (CTCA) has unique diagnostic and prognostic values in cases of giant CAA. Here, we report technical challenges encountered when performed CTCA for a case of Kawasaki disease complicated with giant CAA. In particular, there was significant flow alteration caused by the giant CAA(s) causing suboptimal enhancement when the standard protocol was applied. We share our experience in optimizing the scan and propose the use of either manual bolus tracking or test bolus technique in similar scenarios, as well as multidisciplinary approach to optimize patient preparation.

## Introduction

Kawasaki disease is the most common vasculitis causing acquired coronary artery aneurysm (CAA). Majority of these patients are younger than 5 years old. Echocardiogram is the mainstay of cardiac imaging for Kawasaki disease patients complicated by CAA, but computed tomography coronary angiography (CTCA) has additional diagnostic and prognostic values in giant CAA. Here, we report a case of Kawasaki disease complicated with giant CAA and discuss the technical challenges encountered during CTCA.

## Case report

A 3-month-old baby boy presented to the hospital with fever, bilateral non-purulent conjunctivitis, generalized maculopapular rash, erythematous lips, strawberry tongue, swollen hands and feet, as well as bacille Calmette-Guerin (BCG) scar erythema. Refractory Kawasaki disease was diagnosed as his illness was unresponsive to the initial course of intravenous immunoglobulin (IVIG) treatment. He subsequently required 2 courses of IVIG, one course of intravenous methylprednisolone and oral prednisolone as well as one dose of infliximab for disease control.

Baseline echocardiogram on Day 6 of fever showed normal-sized coronary arteries with right coronary artery (RCA) at 0.9 mm (z score −1.2) and left coronary artery (LCA) at 1.4 mm (z score + 0.77). Dilated RCA was first observed on day 17 echocardiogram, sized at 3.3 mm (z score + 6.08). Subsequent echocardiograms over the following weeks showed rapid progression with development of saccular giant aneurysms over both coronary arteries. Echocardiogram at 26 months of age showed persistent giant saccular aneurysm with LCA at 15 mm (z score +39.09) and RCA at 16 mm (z score +37.46).

**Figure 1.**
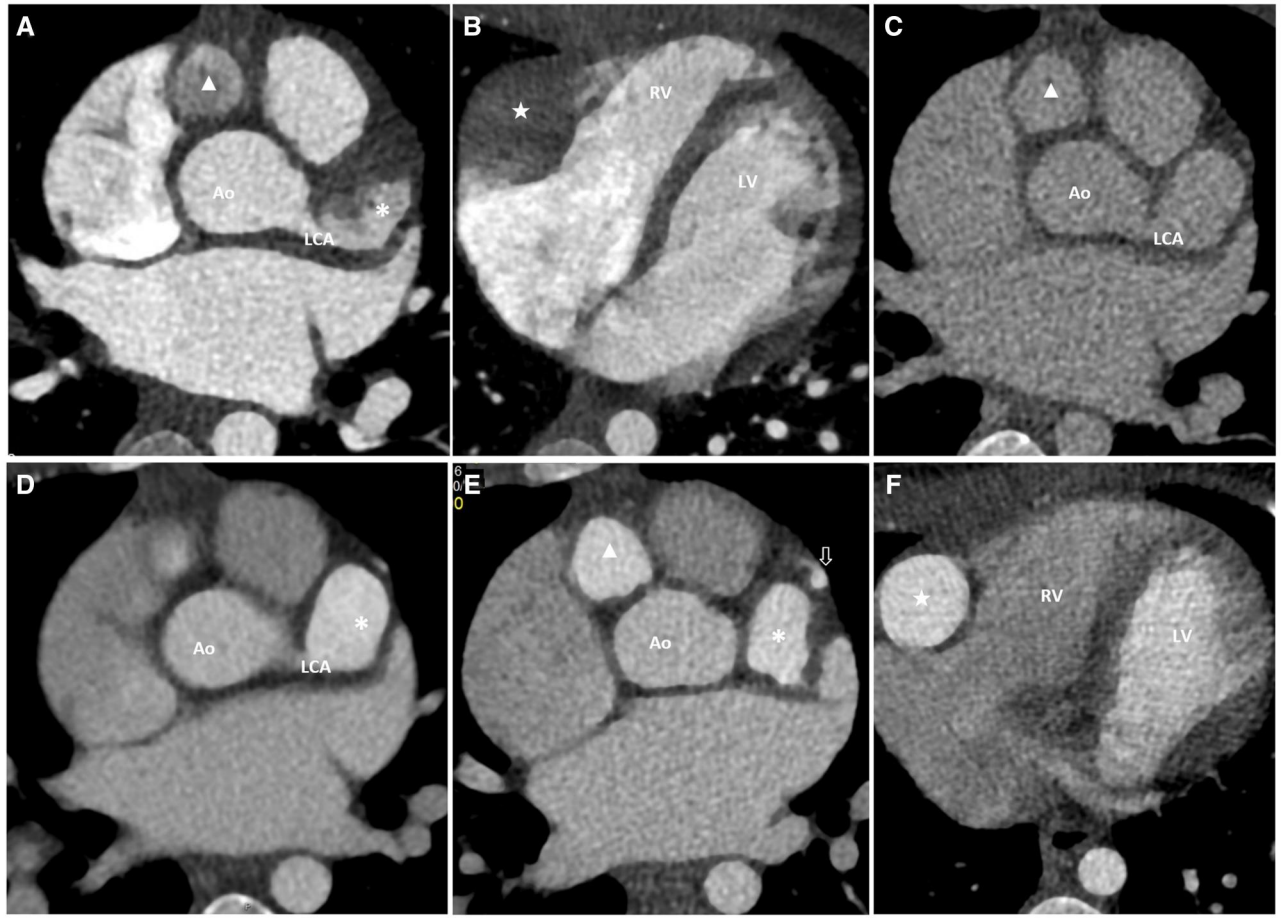
Selected axial images of the contrast-enhanced CTCA acquired in the first episode (A-C) and the second episode (D-F). (A) Axial image during arterial phase in the first baseline CTCA showing only partial opacification in the most proximally located giant aneurysms in LCA (*) and RCA (▲). (B) Axial image from the same scan at a more inferior level showing non-opacification of a mid-RCA aneurysm (★). (C) Venous phase axial image acquired in the first baseline CTCA at the same level as that shown in (A), showing giant aneurysms with suboptimal enhancement. (D-F) After optimization, axial images acquired in the second scan show better contrast enhancement, hence visualisation of the course of the LAD distal to the giant aneurysm (D, E) and multiple aneurysms are identified, including a previously undetected small aneurysm at mid LAD (E, arrow). Abbreviations: RCA = right coronary artery; LAD = left anterior descending artery.

In view of the early age of presentation with refractory disease course and incomplete visualization of the entire course of major coronary arteries by echocardiogram, the first CTCA was arranged when the patient was 30 months old. The study was performed using a dual-source multidetector computed tomography scanner with retrospective electrocardiography gating and reconstructed from 0% to 90% of the R-R interval at 10% intervals. Automatic bolus tracking technique was used with a region-of-interest (ROI) placed at the descending aorta, with a trigger threshold of 80 HU, following our usual practice. The initial acquired images were suboptimal with only the more proximal CAAs partially opacified with layering of contrast within, despite homogenous arterial enhancement in the aorta (Figure 1A and B).

After reviewing the suboptimal images, another set of images was immediately acquired over the same level. Though the more distal CAAs could then be identified, they were not captured with optimal enhancement (Figure 1C). A giant CAA was seen involving the bifurcation into the left anterior descending artery (LAD) and the left circumflex artery, as well as several giant CAAs along the RCA. The LAD distal to the giant aneurysm was not opacified, raising concern for its patency but interpretation was confounded by overall suboptimal contrast enhancement.

After discussion in the multidisciplinary meeting, another CTCA was arranged 2 months later. To optimize the imaging, we made 2 major changes to our scanning technique: (1) In the baseline scan, which was suboptimal, although our patient was premedicated with a low dose of beta-blocker, his heart rate remained relatively fast during the examination (average heart rate 99-101 beats per minute [bpm]). For maximal technical optimization of the second scan, a beta blocker was titrated until the patient’s heart rate was maintained between 60 and 70 bpm. (2) Instead of using automatic bolus tracking, we adopted manual bolus tracking in the second scan. Scanning was started when homogeneous opacification of the most proximal RCA aneurysm was observed.

In the follow-up scan, there was significantly improved opacification of the CAAs. The course of the LAD distal to the giant aneurysm could now be delineated (Figure 1D–F). Two additional small saccular aneurysms could be identified at mid-LAD. Overall the CAAs showed no interval change in size compared with the previous scan, measuring up to 11 mm at LCA (Z-score 26.75) and 17 mm at RCA (Z-score 39.02). No mural thrombus was detected.

**Figure 2.**
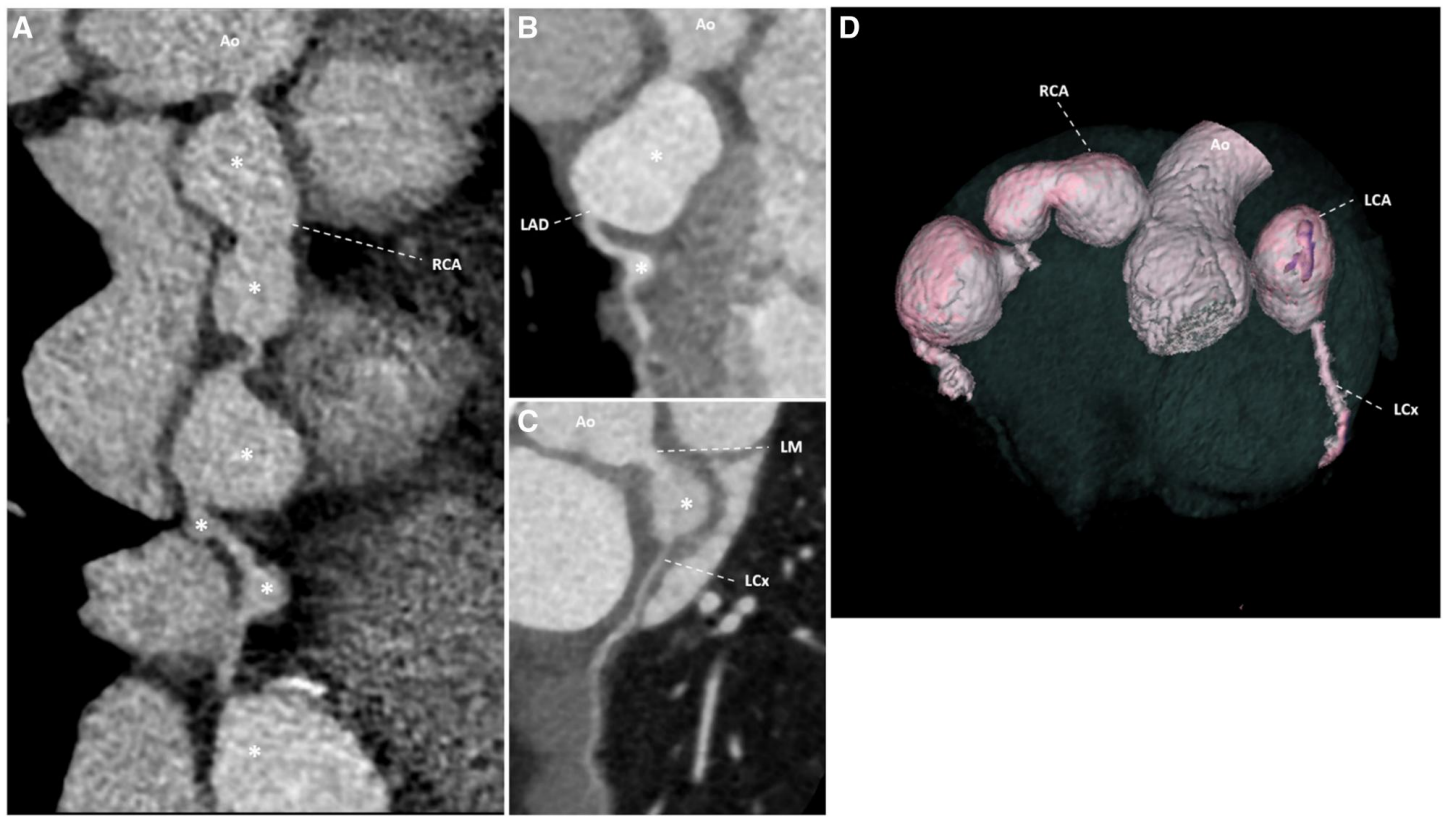
Contrast-enhanced retrospectively ECG-gated dual-source coronary CT angiography in a 30-month-old boy with history of Kawasaki disease in infancy. Curved multiplanar reformation of the (A) RCA, (B) LAD, and (C) LCx demonstrates multiple saccular aneurysms (*). Four of the RCA aneurysms in (A) meet the criteria for giant CAA. Along the LCA (B), there is a giant aneurysm involving distal LM and proximal LAD and a mild aneurysm in mid LAD. The origin of LCx (C) is involved by a giant aneurysm. (D) Three-dimensional volume rendering shows multiple giant CAAs. Abbreviations: RCA = right coronary artery; LAD = left anterior descending artery; LCx = left circumflex coronary artery.

3D-reconstructed images were obtained with volume rendering technique so that distribution of the aneurysm along the CAAs can be better appreciated (Figure 2).

## Discussion

Kawasaki disease is an acute self-limiting vasculitis of unknown aetiology affecting infants and young children. Its exact aetiology remains unknown, but there are important genetic contributions to disease susceptibility and postulations of immune system responses activated by an inhaled antigen.^1^ Medium-sized extra-parenchymal muscular arteries, most commonly the coronary arteries, are affected.

CAA is defined as a dilated vessel segment of at least 1.5 times larger in diameter than the adjacent normal arterial segments. By the Japanese criteria, CAAs are further classified according to internal lumen size: small, ≤4 mm; medium, >4 to ≤8 mm; and giant, >8 mm.^2^ As these criteria does not account for patient size, various Z-score methods for normalizing coronary artery luminal dimensions are developed. Classification scheme based on Z-scores is preferred and a Z-score ≥10 is classified as giant aneurysm.^3^

CAAs develop in 15%-25% of children not treated with IVIG during the febrile phase of Kawasaki disease. Even with treatment, 5%-10% of patients develop one or more CAA(s).^4^ Risk factors include male gender, aged younger than 6 months old or older than 8 years, significant systemic inflammation (evident by laboratory tests),^5^ and resistance to IVIG treatment.^6^ Our patient belonged to the vulnerable group with several of the above-stated risk factors.

Aneurysms are usually detected within 10-14 days of disease onset and may increase in size over the first 2 months. Mild and moderate CAAs generally follow a benign natural course with the former regressing within a short time and the latter eventually reducing in size in most cases. In contrast, giant CAAs are commonly persistent and may progress to obstructive or stenotic lesions.^7^ Giant CAAs with bilateral coronary artery involvement pose the worst prognosis. According to a previous study, at 30 years the survival rate and cardiac event-free rate are 87% and 21%, respectively, while up to 69% of patients had undergone coronary artery bypass graft.^8^ It is observed that the course of coronary artery disease depends on the size and distribution of aneurysms at initial angiography.^7^ A comprehensive, quantitative assessment at initial presentation therefore carries useful prognostic value.

Echocardiogram is the mainstay of cardiac imaging during the acute phase, offering both functional assessment and detection of coronary artery dilatation. It is readily available and radiation free, suitable for repeated assessments at 1-2 week intervals. However, it is operator-dependent with lower sensitivity and limitation in detection of obstructive and stenotic coronary lesions. Comparatively, CTCA is superior for the identification of fusiform and distally located aneurysms.^9^ In our case, the baseline echocardiogram already detected giant aneurysms. As the child grows in body size, the coronary arterial system, in particular the distal segments, cannot be fully assessed by echocardiogram. In this case, we demonstrate that CTCA can detect more aneurysms than previously seen on echocardiography. Furthermore, CTCA has better depiction of any layering intraluminal thrombus and overall vessel patency. The advantages of CTCA over cardiac catheterization include less invasiveness, as well as demonstrating the true sizes of aneurysms rather than only providing endoluminal views. Isotropic resolution of CTCA also facilitates accurate 3D reconstruction and structural visualization.

To achieve high-quality arterial phase image acquisition, important technical factors to be considered include contrast volume and injection rate. The maximal volume of contrast that can be injected is determined by body weight, whereas the maximal injection rate is limited by catheter size. In this small child, we use 2 mL of Omnipaque 350 per kilogram of body weight, hence a total volume of 28 mL, followed by a 30-40 mL saline flush. For adult CTCA, the standard practice is an injection rate of 4-5 mL/s via a 16G catheter. In our case, we achieved an injection rate of 2.3-2.5 mL/s via a 22G catheter. Although numerically it is half the usual injection rate in adults, the difference is likely off-set by the overall smaller blood pool and cardiac output in a child patient. During both CTCA studies, sufficiently sharp rises in arterial enhancement within the descending aorta were achieved during real-time monitoring.

An unexpected suboptimal opacification of the CCA was encountered in the first scan. The presence of multiple giant aneurysms likely caused significant alteration to the flow dynamics of both coronary arteries. Under normal circumstances, even in severe adult coronary atherosclerotic disease, the coronary arteries reach peak contrast enhancement shortly after that in the descending aorta, hence the descending aorta is often used as the ROI for bolus tracking. Unfortunately, in our patient, there was significant delay in the opacification of the coronary arteries. Gaining from the experience of the suboptimal initial scan, we have tailor-made technical alternation in subsequent scan. With this experience, we propose that if there is significant disease burden in both coronary arteries leading to unpredictable bolus timing, it may be advisable to adopt manual bolus tracking with the ROI placed outside the scanning zone in the air and scanning should be start manually when the operator observes opacification in the CCA(s) An alternative method would be the test bolus technique, in which a smaller bolus of contrast is injected prior to the actual study and time-dependent contrast enhancement curve of the selected ROI is obtained to guide the timing of the image acquisition. The latter, however, may be less desirable as it necessitates additional contrast injection and radiation dose to the patient.

Heart rate control is important to reduce blurring artefacts and radiation in CTCA. Target heart rate is generally ≤65 bpm in adults. In the second CTCA study, we have adopted a multidisciplinary approach. Our young patient was closely monitored by a clinical team consisting of experienced paediatrician and nurses during the examination. Patient was given beta blocker in phases achieving a target heart rate of 60-70 bpm for the study (baseline 90-100 bpm). The team has prepared atropine injection standby as antidote in case any adverse events encountered. Compared with adults, children are generally more prone to bradycardia, hence we proceeded only as our case’s condition allowed under close monitoring. Their baseline blood pressures are also generally lower, which may be another concern for premedication with beta blockers. As the development of high-specification CT scanners (eg, dual-source, >64-slice scanners) permits higher image quality with higher heart rates, satisfactory image quality is achievable at higher heart rate of 85-90 bpm and this could serve as an appropriate target heart rate in children.

Image quality significantly improved in the second scan after the above adjustments to the CTCA scanning protocol, which was proven to be useful for interpretation of the scan findings.

## Conclusion

CTCA has an important role in assessment of giant CAAs in Kawasaki disease, offering higher detection sensitivity and quantitative assessment of the size and distribution of aneurysms (ie, both key prognostic factors), as well as better depiction of intramural thrombus and stenotic coronary lesions, which are dreaded complications affecting long-term prognosis. This case report sought to share our experience of technical modifications in optimizing CTCA for the assessment of giant CAAs in paediatric patients.

## Learning points

- CAA develops in 5%-10% of cases with Kawasaki disease despite treatment. Mild and moderate CAAs generally have a benign disease course, while giant CAAs are commonly persistent and may progress to obstructive or stenotic lesions, associated with considerable morbidities. A quantitative assessment at initial presentation carries useful prognostic value.
- CTCA is superior for detection of fusiform, distally located CAAs and assessment of intraluminal thrombus and vessel patency.
- In presence of giant CAAs, there may be significant flow alteration. When performing CTCA in these cases, it is advisable to adopt manual bolus tracking or test bolus technique and multidisciplinary approach to optimize patient preparation.

## References

[1] Rodó X , Curcoll R, Robinson M, et al Tropospheric winds from northeastern China carry the etiologic agent of Kawasaki disease from its source to Japan. Proc National Acad Sci USA. 2014;111(22):7952-7957. 10.1073/pnas.1400380111 PMC4050536 24843117

[2] Fukazawa R , Kobayashi J, Ayusawa M, et al; Japanese Circulation Society Joint Working Group. JCS/JSCS 2020 guideline on diagnosis and management of cardiovascular sequelae in Kawasaki disease. Circ J. 2020;84(8):1348-1407. 10.1253/circj.cj-19-1094 32641591

[3] McCrindle BW , Rowley AH, Newburger JW, et al; American Heart Association Rheumatic Fever, Endocarditis, and Kawasaki Disease Committee of the Council on Cardiovascular Disease in the Young; Council on Cardiovascular and Stroke Nursing; Council on Cardiovascular Surgery and Anesthesia; and Council on Epidemiology and Prevention. Diagnosis, treatment, and long-term management of Kawasaki disease: a scientific statement for health professionals from the American Heart Association. Circulation. 2017;135(17):e927-e999. 10.1161/CIR.0000000000000484. Erratum in: Circulation. 2019;140:e181–e184. 28356445

[4] Newburger JW , Takahashi M, Burns JC. Kawasaki disease. J Am Coll Cardiol. 2016;67(14):1738-1749. 10.1016/j.jacc.2015.12.073 27056781

[5] McCrindle BW , Li JS, Minich LL, et al; Pediatric Heart Network Investigators. Coronary artery involvement in children with Kawasaki disease: risk factors from analysis of serial normalized measurements. Circulation. 2007;116(2):174-179. 10.1161/circulationaha.107.690875 17576863

[6] Uehara R , Belay ED, Maddox RA, et al Analysis of potential risk factors associated with nonresponse to initial intravenous immunoglobulin treatment among Kawasaki disease patients in Japan. Pediatric Infect Dis J. 2008;27(2):155-160. 10.1097/inf.0b013e31815922b5 18174868

[7] Nakano H , Ueda K, Saito A, Nojima K. Repeated quantitative angiograms in coronary arterial aneurysm in Kawasaki disease. Am J Cardiol. 1985;56(13):846-851. 10.1016/0002-9149(85)90767-2 4061324

[8] Tsuda E , Hamaoka K, Suzuki H, et al A survey of the 3-decade outcome for patients with giant aneurysms caused by Kawasaki disease. Am Heart J. 2014;167(2):249-258. 10.1016/j.ahj.2013.10.025 24439987

[9] Chu WC , Mok GC, Lam WW, Yam MC, Sung RY. Assessment of coronary artery aneurysms in paediatric patients with Kawasaki disease by multidetector row CT angiography: feasibility and comparison with 2D echocardiography. Pediatr Radiol. 2006;36(11):1148-1153. 10.1007/s00247-006-0281-4 16912893

